# Supplementation of dietary germanium biotite enhances induction of the immune responses by foot-and-mouth disease vaccine in cattle

**DOI:** 10.1186/s12917-014-0179-6

**Published:** 2014-08-12

**Authors:** Myunghwan Jung, Min-Kyoung Shin, Seung-Bin Cha, Seung Won Shin, Anna Yoo, Won-Jung Lee, Hong-Tae Park, Jong-Hyeon Park, Byounghan Kim, Yeon-Kwon Jung, Han Sang Yoo

**Affiliations:** 1 Department of Infectious Diseases, College of Veterinary Medicine, Seoul National University, Seoul 151-742, South Korea; 2 Foot and Mouth Disease Division, Animal and Plant Quarantine Agency, Anyang 430-757, Gyeonggido, South Korea; 3 Seobong BioBestech Co., Ltd, Hyechon Bldg. #401, 831 Yeoksam-dong, Kangnam-gu 135-792, Seoul, South Korea; 4 Institute of Green Bio Science and Technology, Seoul National University, Pyeongchang 232-916, South Korea

**Keywords:** FMD, Germanium biotite, Immune responses, Cattle

## Abstract

**Background:**

After the recent outbreak of foot-and-mouth disease (FMD) in Korea, a vaccination policy has been applied to control the disease. In addition, several non-specific immune stimulators have been used without any scientific evidence that they would enhance the immune response after FMD vaccination and/or protect against FMD. Based on the current situation, the aim of this study was to evaluate the effect of the non-specific immune stimulator germanium biotite on FMD vaccination and immune responses in cattle. To achieve our goal, immune responses to FMD vaccination, such as levels of IgG and IgA, antibody duration, and virus-neutralizing titers were investigated after germanium biotite feeding. The PBMC typing and proliferative response after stimulation with mitogens, the cytokines expression level of PBMC, and the lysozyme activity in the serum were measured to evaluate the immune enhancing effects of germanium biotite following its administration.

**Results:**

Following the first vaccination, high level of IgG (at 4 weeks) and IgA (at 2 and 31 weeks) titers in serum and saliva were observed in the germanium biotite-feeding group (*p* < 0.05). The germanium biotite group also showed high and longstanding inhibition percentage value in ELISA assay at 31 weeks (*p* < 0.05). Generally, higher virus-neutralizing antibody titers were observed in the feeding group at 20 and 31 weeks after vaccination. Following the feeding germanium biotite, the germanium biotite group showed increased subpopulation of CD4^+^ lymphocytes and MHC I^+^II^+^ cells in PBMCs at 23 week, responding to stimulation of ConA. The levels of IFN-γ (at 3 and 8 weeks), IL-1α (at 3, 11, and 23 weeks), IL-1β (at 3, 8, and 11 weeks), and IL-4 (at 8 and 11 weeks) gene expression were also significantly increased in the feeding group (*p* < 0.01 and *p* < 0.05). Feeding with germanium biotite increased the lymphocytes’ proliferative response to the stimulation of ConA and LPS at 23 weeks and lysozyme activity at 9 weeks after feeding.

**Conclusions:**

These results suggest that germanium biotite feeding could increase the protection against FMD virus infection via the induction of higher humoral and cellular immune responses in cattle.

## 1 Background

Foot-and-mouth disease (FMD) is a highly contagious and economically important disease that affects cloven-hoofed animals, and it is characterized by appetite loss, an increase in body temperature, and vesicles in the mouth, tongue, hooves, and nipples [1],[2]. The disease is caused by the FMD virus (FMDV), which is a small, icosahedral, non-enveloped RNA virus classified within the *Aphthovirus* genus as a member of *Picornaviridae*. The clinical severity of the disease varies with the strain of FMDV, infection dose, species, and individual susceptibility of the host [3]. The disease can be transmitted via direct or indirect contact between FMDV-infected animals and susceptible animals [2].

Due to its high mutation rate, this virus exists as seven distinct serotypes (O, A, C, Asia 1, South African territories [SAT]1, SAT2, and SAT3) as well as numerous and constantly evolving subtypes, which shows a spectrum of antigenic diversity [2],[3]. Therefore, one major problem in controlling FMD is antigenic variation, as infection or vaccination with one FMDV serotype does not protect against other serotypes, and it may even fail to protect fully against other subtypes within the same serotype [4]–[6]. This problem has been raised by the experimental and field data of previous researchers on vaccination, including both single and multivalent vaccines [3],[7],[8].

Despite the powerful effects of vaccines, which have mitigated enormous FMD outbreaks, the current vaccines have many problems to overcome such as the long time required to induce antibodies and short duration of immunity when applying them to emergency and routine vaccination programs [6]–[9]. Therefore, strategies to improve the immune response to vaccination have included using higher vaccine doses or increasing the number of doses, using different routes of administration, accelerating the dosing schedule, and using adjuvants such as antigen delivery systems and various immunostimulators [10].

The immune response against FMDV is related to circulating humoral antibody titers; this is considered the most important factor in conferring protection against FMD [4]. The importance of cell-mediated immunity also has been recognized in the induction of humoral immunity and the clearance of FMDV. T-cell responses mediated by CD4^+^ cells are required for protective immunity against FMDV as they participate in the production of antiviral antibodies [11]. Subsets of the CD4^+^ major histocompatibility complex (MHC) class II-restricted T-cells respond to activation by antigen-presenting cells and antigens by producing a T helper type 1 (Th1, IFN-γ) and T helper type 2 (Th2; IL-4, IL-5, and IL-13) responses [12]. Several studies have also demonstrated the presence of FMD-specific MHC II-restricted responses in cattle and pigs [11]–[13]. The antiviral responses of CD8^+^ T-cells were also detected following FMDV infection in previous studies [14],[15]. These antiviral responses were through direct cytotoxicity or release of cytokines such as IFN-γ following vaccination, and that the antiviral responses were almost 100 times higher following re-stimulation [15]. MHC class I-restricted CD8^+^ T-cells also showed specific immune response to FMDV as memory cells; however, the correlation between FMDV-specific CD8^+^ T-cell recognition and protection remains to be defined [14],[15]. In addition, the innate immune systems, as well as adaptive immune, play an important role in immune responses to FMDV infection [16],[17]. The innate immune system, characterized by non-specific responses, is associated with early protection against FMD and is involved in the formation of adaptive immune response to FMDV infection [16],[17].

Germanium biotite, as a well-known feed supplement, is a common phyllosilicate mineral that contains potassium, magnesium, iron, aluminum, and silicate. It has been reported that the effects of non-specific immune stimulating of biotite are associated with the immune cells being stimulated by silicates [18]–[20]. Fibrogenic silicates (SiO_2_) activated proinflammatory macrophages, and aluminosilicates (Al_2_SiO_5_) improved immune-cell differentiation [21]. Aluminosilicates act as a nonspecific immunostimulator that is similar to a superantigen [22]. Superantigens are a class of extremely potent T-cell mitogens, and have a high affinity to regions of MHC II molecules [23]. Indeed, proinflammatory macrophages, which belong to MHC class II antigen–presenting cells, are activated by fibrogenic silicate particulates [21]. Thus, previous studies have suggested that germanium biotite has a potential as a new supplement for immunostimulators, prophylactic agents, and remedial agents [20].

Since the devastating FMD outbreak in Korea at the end of 2010, cattle, pigs, and some small ruminants have been vaccinated with a trivalent FMD vaccine (O1 Manisa, A Malaysia 97, and Asia1 Shamir) at least 6 protective dose 50% (PD_50_). [24]. To improve the vaccine efficacy, several non-specific immune stimulators have been used in Korea without any scientific understanding of their effects. Therefore, as a first step to understanding the immunological mechanism and improve the vaccine’s efficacy, we examined the effects of a non-specific immune stimulator, germanium biotite, in relation to the FMD vaccine in cattle.

## 2 Results

### 2.1 Antibody responses to FDMV

**Figure 1:**
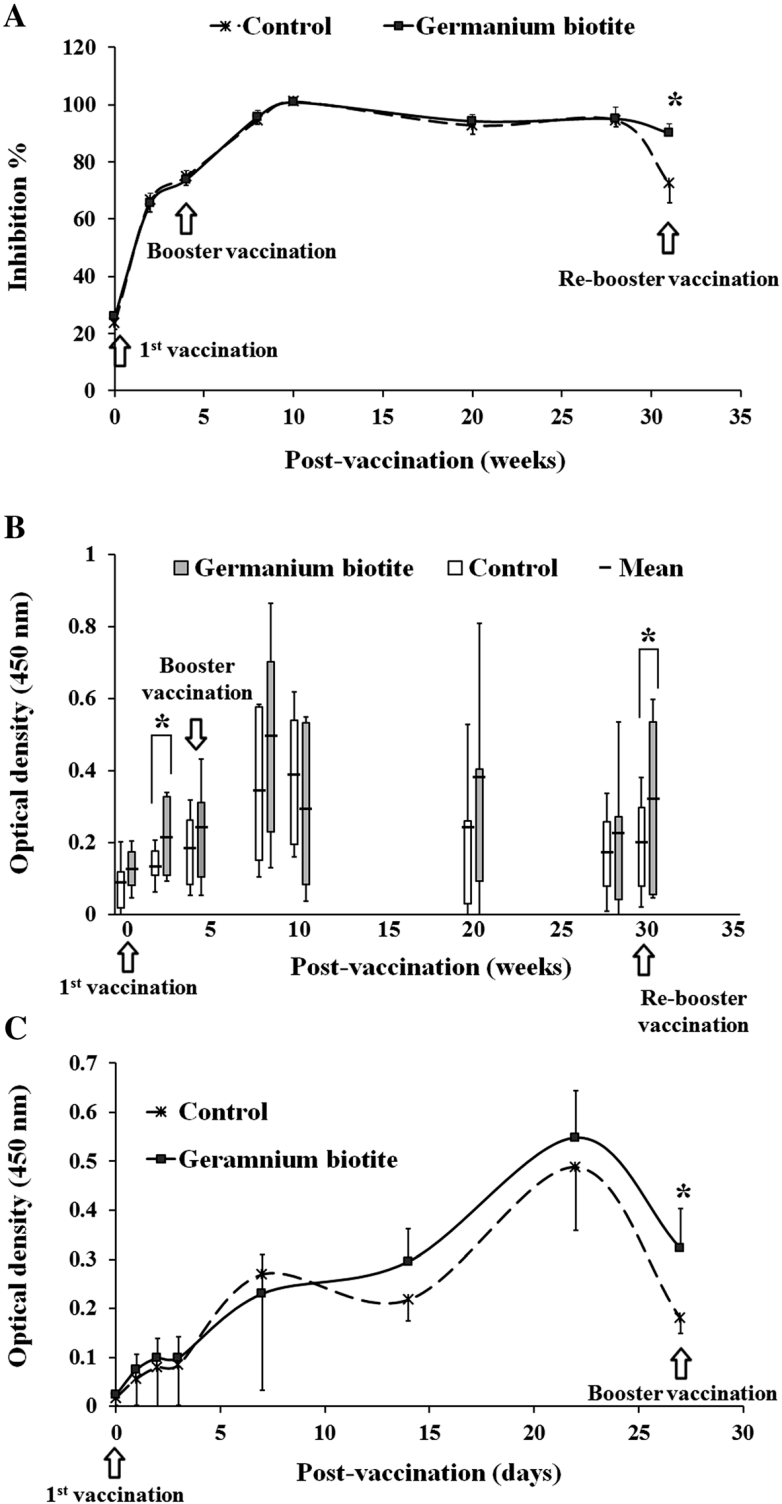
**Humoral immune responses after vaccination with Decivac FMD DOE trivalent vaccine in cattle. A**. Inhibition percentage (PI) of the antibody against FMDV serotype O in both the germanium biotite-feeding group and the non-feeding group after vaccination. The high PI value indicates a high level of antibody titer, and the sera that showed more than 50% of the PI value were considered to be seropositive in the ELISA assays. **B**. The FMDV serotype O-specific IgA response in the saliva of cattle of the germanium biotite and the control groups after vaccination with Decivac FMD DOE trivalent vaccine. **C**. Induction of FMDV serotype O-specific IgG in the cattle of the germanium biotite and control groups after vaccination with Decivac FMD DOE trivalent vaccine.

In the analysis of the duration of antibody levels and the secretion of IgA after FMD vaccination, the inhibition percentage (PI) values started to increase with the first vaccination, and the values were significantly increased by a booster vaccination at 4 weeks, with the highest PI values at 10 weeks, and this continued steadily up to 28 weeks, regardless of whether the germanium biotite was administered. However, the values decreased at 31 weeks in the control group when the re-booster vaccination was required, while the values continued at 31 weeks in the germanium biotite group (Figure 1A).

FMDV-specific IgA in the saliva of the germanium biotite group was generally higher than in the control group, although there were no significant differences between the groups at some points during this experiment (Figure 1B). However, IgA could not be detected in the feces of the two groups with the kit used (data not shown).

In the analysis of the early response of IgG, both groups showed a steady increase in the IgG antibody level up to 3 weeks after vaccination; after this point, the level decreased (Figure 1C). The IgG level in the germanium biotite group was higher than in the control group, although the difference was not significant. However, there was a significantly higher IgG antibody level at 4 weeks after vaccination when the booster vaccination was required (*p* < 0.05, Figure 1C).

### 2.2 Virus neutralization antibody against the heterologous FMDV strain

As shown in Table 1, the sera of the control group had a titer of less than 16 at both points. On the other hand, while the germanium biotite group had a VN titer of 16 or less at 20 weeks after vaccination, the VN titer in the group was found to have increased at 31 weeks after vaccination.

**Table 1:** Virus neutralization antibody titers in sera after vaccination

**Group**	**Samples**	**20 weeks**	**31 weeks**
Control	1	<16	<16
	2	<16	<16
	3	<16	<16
	4	<16	<16
	5	<16	<16
	6	<16	<16
	7	<16	<16
Germanium biotite	1	<16	16
	2	16	<16
	3	16	32
	4	16	26
	5	16	<16
	6	16	<16
	7	<16	26
	8	<16	32

The selected sera were taken from the same cattle in each collection date. All cattle were 6-month-old when the first vaccination was conducted. The sera selected from the germanium biotite group show a higher virus neutralization antibody titer than the control group.

### 2.3 Analysis of peripheral blood mononuclear cell subpopulation

Changes in the subpopulations of five different lymphocytes were analyzed based on the administration of the germanium biotite. At 23 weeks, CD4^+^ lymphocytes and MHCI^+^II^+^ cells in the germanium biotite group were significantly higher than in the control group (*p* < 0.01), while CD79a^+^, CD3^+^, and CD8^+^ lymphocytes exhibited no differences between the two groups (Table 2).

**Table 2:** Subsets of peripheral blood mononuclear cells after mitogen stimulation

	**Before administration**		**23 weeks after administration**		**Stimulators**
	**Control**	**Germanium biotite**	**Control**	**Germanium biotite**	
CD79a^+^	8.40% (±0.17)	7.90% (±0.14)	6.70% (±0.53)	4.70% (±1.01)	LPS
CD3^+^	59.50% (±0.49)	57.80% (±0.53)	93.50% (±0.04)	93.10% (±0.28)	ConA
CD4^+^	14.10% (±0.72)	13.00% (±0.22)	15.40% (±0.58)	23.80% (±0.52)**	ConA
CD8^+^	12.50% (±0.74)	10.40% (±1.30)	14.30% (±1.40)	10.20% (±1.21)	ConA
MHC Ι^+^, ΙΙ^+^	43.00% (±0.45)	37.50% (±0.12)	8.80% (±0.17)	10.20% (±0.15)**	ConA

The data were expressed as mean ± standard deviation (SD) (**p < 0.01 compared to the control group at each time point).

### 2.4 Analysis of cytokine gene expression

**Figure 2:**
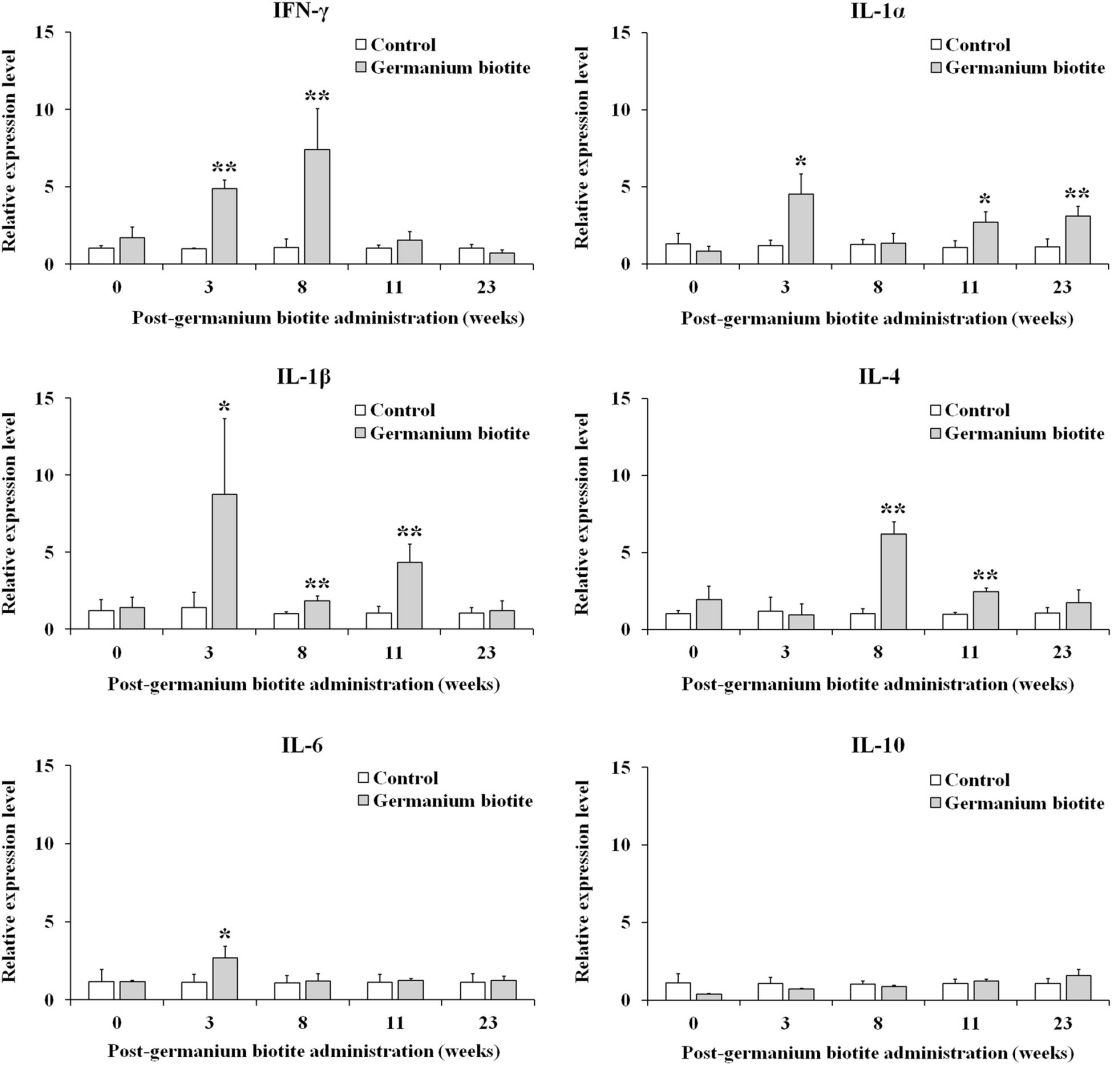
**Analysis of gene expression of cytokines IFN-γ, IL-1α, IL-1β, IL-4, IL-6, and IL-10.** Total RNA were purified from peripheral blood mononuclear cells separated from cattle vaccinated and fed with the germanium biotite supplement or without the supplement.

The gene expression of IFN-γ, IL-1α, IL-1β, and IL-4 was significantly higher in the germanium biotite group during this experiment (Figure 2). The significant high levels of IFN-γ were determined in the germanium biotite group at 3 and 8 weeks, compared to the control (*p* < 0.01). In case of IL-1α and IL-1β, significant differences were observed at 3, 11, and 23 weeks and at 3, 8, and 11 weeks, respectively (*p* < 0.05 and *p* < 0.01). The germanium biotite group also showed significant high levels of IL-4 at 8 and 11 weeks, compared to the control (*p* < 0.01). However, there were no noticeable differences in the expression levels of IL-6 and IL-10 between the two groups although a significant difference in IL-6 expression was observed at 3 week. The germanium biotite feeding induced a significant increase in IFN-γ gene expression at a relatively early stage and in IL-4 at a late stage, while increases in IL-1α and IL-1β gene expression were observed throughout the experimental period.

### 2.5 Lymphocyte proliferation

**Figure 3:**
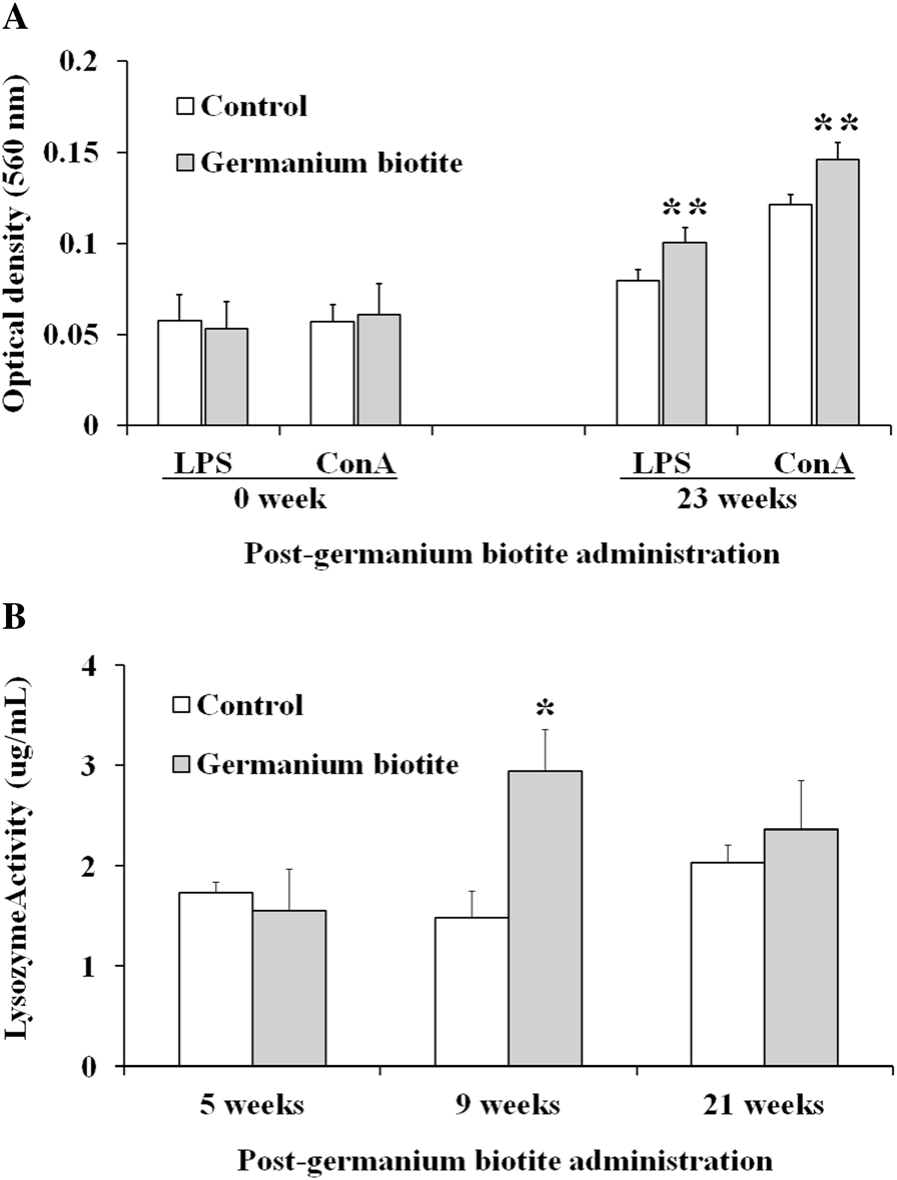
**Proliferation of peripheral blood mononuclear cells (PBMCs) and lysozyme activity of sera. A**. PBMCs were separated from the vaccinated cattle that were fed with the germanium biotite supplement or without the supplement at 0 and 23 weeks. The proliferation of the PBMCs was estimated after stimulation with LPS or ConA. **B**. The lysozyme activities of the sera from the vaccinated cattle that were fed with the germanium biotite supplement or without the supplement were measured based on the dissolution ability against *Micrococcus lysodeikticus*.

Higher lymphocyte proliferation in the germanium biotite group was observed by LPS and ConA stimulation at 23 weeks compared to the control (Figure 3A, *p* < 0.05).

### 2.6 Lysozyme activity

Of the three points at which the lysozyme activity was measured in the serum, significantly higher lysozyme activity was observed in the germanium biotite group at 9 weeks after starting the feeding (Figure 3B, *p* < 0.05). The increase was observed at a relatively early stage after germanium biotite feeding.

## 3 Discussion

The current FMD vaccines that are used in cattle need to be administered every 6 months, in accordance with the duration of the antibody, to protect against the disease [5],[6],[25],[26]. The diversity of FMD serotypes has also raised issues related to the efficacy of the current FMD vaccines. Furthermore, there is considerable argument about whether the trivalent vaccines show protective abilities against the Andong strain through the cross-reactivity of antibodies induced by trivalent vaccination [24]. Based on the current knowledge of FMD vaccines, the need for additional materials and/or methods has been raised with a view to overcoming the problem. Therefore, non-specific immune stimulators have received attention as a possible solution to this issue. In the disastrous FMD outbreak in Korea in 2010 [24], many non-specific immune stimulators were used without any scientific evidence that they would enhance protection against FMDV infection. Although non-specific immune stimulators have been used, their efficiency is still controversial. Therefore, we evaluated the effect of a non-specific immune stimulator, germanium biotite, for FMD vaccination in cattle.

Previous studies reported that a re-booster vaccination was needed 6 months after the booster vaccination based on the decrease of antibody titer against FMDV [5],[6],[25],[26]. Coinciding with these previous studies, this study observed a decreased PI value in the control group at 31 weeks. However, the germanium biotite group showed a continued high level of PI value for 31 weeks. In order to elevate the antibody level, a booster vaccination was needed 4 weeks after the first vaccination. At that time, the germanium biotite group showed a high level of IgG antibody compared to the control. This experimental result closely matches with previous studies’ findings on the improvement of antibody production by germanium biotite [18],[19]. Furthermore, germanium biotite feeding showed a high level of IgA in the saliva, which could provide a beneficial effect when it comes to protecting against FMDV infection in cattle [27]. In addition, the IgG response suggested that the point at which to administer the booster injection in the FMD vaccine program for cattle is the same as indicated in previous studies [6],[26]. However, the increase in antibody duration suggests that the current FMD vaccine program should be reconsidered in animals fed germanium biotite. In this study, sampling of sera after 31 weeks from only first and boosting vaccinated cattle was limited, due to national FMD control policy by which re-booster vaccination carried out every 6 months in cattle. The decline point of PI 6 month after booster vaccination could not be identified. Therefore, the investigation of PI value changes for a longer period should be conducted in the future study to determine the re-booster vaccination timing.

Along with these antibody responses, a VN test was performed using the Andong strain, which is an isolate from the recent outbreak in Korea in 2010. Following the vaccination, 20 weeks was the mid-point of the period showing a high PI value and 31 weeks was the time point that showed a difference in PI values between the two experiment groups. To ensure the reliability of VN test and reduce the variation, cattle in Farm B were selected and sera of half the number of scale of each group in Farm B were taken from the same cattle in each collection date. As shown in Table 1, all experimental groups showed a low VN titer. These low VN titers might be due to using the heterologous FMDV. The immunological relationship between the O1 Manisa vaccine and the Andong strains was relatively low or moderate (*r*-value of approximately 0.3) [28]. A previous study considered an *r*-value in the range of 0.3–1.0 as indicative of reasonable levels of cross-reactivity [29]. Therefore, the low VN titers observed in this study could be explained by the low level of the *r*-value between the two strains. However, in comparison with the control group, the increase of VN antibodies in the germanium biotite group suggests that protectivity against FMDV infection might be increased through the supplementation of germanium biotite [3],[6],[9].

Although the importance of antibodies in protection against FMDV infection is well known [3],[6],[9], a specific antibody does not guarantee sterile immunity or clinical protection against FMDV infection [9],[25]. Moreover, protection against FMDV infection has been observed in the absence of a detectable specific humoral response [30]. Therefore, it is reasonable to consider a possible protective role for innate immune responses in FMD infection and for control of the disease [12],[13],[26],[31]. In this study, high lysozyme activity—indicating macrophage activity—was observed in the germanium biotite group compared to the control group. This result corresponds with the findings of previous studies, which that the macrophage activation effects of silicate (SiO_2_) and aluminosilicate (Al_2_SiO_5_), which are major components of germanium biotite [18],[19],[21]. Along with macrophage activation, a high proliferative response of T and B lymphocytes to the mitogen stimulation was observed in the germanium biotite group compared to the control group, thereby indicating that the marker level of lymphocyte activity in the germanium biotite group was higher than in the control group. Through the analysis of PBMC subset stimulation with ConA and LPS, it can be presumed that the proliferative responses of the T lymphocytes were predominately associated with CD4^+^ and MHCI^+^ II^+^ cells, as the CD4^+^ and MHCI^+^ II^+^ cells were significantly increased at 23 weeks after stimulation with ConA. The important role of CD4^+^ T-cells in the induction of the antibody response in ruminants following infection or vaccination with a virus or a viral peptide has been demonstrated [11],[12]. Furthermore, MHC II–restricted T-cells (CD4) play an important role in the immune responses to the FMDV antigen and the activation of macrophages [31]. These results show the increase of activation markers for innate immune responses associated with cell-mediated immunity in the germanium biotite group. In addition, previous studies reported that the cross-reactivities against a heterologous virus were mainly induced by T-cell responses to FMDV vaccination [13]. Therefore, it could be postulated that the increase of VN titer against Andong strain in the germanium biotite group compared to the control might result from enhanced T-cell responses due to germanium biotite feeding.

The increase of the expression of the IFN-γ, IL-1α, IL-1β, and IL-4 genes in the germanium biotite group was in agreement with the results that demonstrated the activation of immune cells associated with cell-mediated immune responses. The increase of the IFN-γ expression level and the CD4^+^ T-cell subpopulation in our results is closely correlated with a previous report that indicated that CD4^+^ T-cells were the major proliferating phenotype and IFN-γ producing cells [12]. The induction of IFN-γ expression has been observed in antigen-specific T-cell activation [32], MHC II–restricted T-cells (CD4) [5], and activation of macrophages [31]. This immune cell activation induced by IFN-γ expression could also be observed in our results through the increases in the CD4^+^ and MHCI^+^ II^+^ cell subpopulations, lysozyme activity, and lymphocyte proliferation level. In addition, the antiviral activity of IFN-γ against FMDV contributed to the control of FMDV replication and the spread of the virus within the host via activation of NK cells and macrophages [33]. IL-1α and IL-1β are mainly produced in activated macrophages and promote Th2 immune cells, which represent the major source of IL-4 [34]. IL-4 also promotes Th2 cell growth and enhances MHC II expression in B-cells [34]. These results suggest that germanium biotite induces Th1 and Th2 responses through the activation of macrophages and CD4+ class II MHC–restricted T-cells [1],[34]. Considering these results, it can be presumed that supplementation of germanium biotite may activate cell-mediated immunity, thereby enhancing the induction of the immune responses by FMD vaccination and the protectivity against FMDV infection. However, the effects of germanium biotite on T-cell activation through FMDV specific T-cell responses could not be investigated. Therefore, an inquiry on the effects of germanium biotite on immune responses to FMDV vaccination through a comparative study of T-cell responses using FMDV antigen were needed to evaluate the specific effects in a further research.

In addition, a large difference in the appearance of the MHC II subpopulation was observed in the experiment cattle between two time points, before and after the administration of the germanium biotite. Previous studies showed that the MHC II subpopulation in the PBMCs of Holstein at 5–7 and 15–16 months could be estimated at about 32.2% and 10.7%, respectively [35],[36]. Based on these reports, it can be presumed that the significant difference in this study could be caused by the age change in the experiment calves.

## 4 Conclusions

The results of this study suggest an enhancement of the immune responses to FMD vaccination through the supplementation of dietary germanium biotite such as IgG, IgA, and VN antibodies. It can be presumed that these enhancements are induced by the immunostimulating effects of the germanium biotite, including the activation of macrophages and CD4+ class II MHC–restricted cells, as well as the induction of cytokines, which can activate Th1 and Th2 immune cells. In conclusion, the findings of this study indicate that dietary germanium biotite could be used to improve the efficacy of the FMD vaccine.

## 5 Methods

### 5.1 Experimental animals and vaccination

This experiment was carried out with 89 cattle from 6–8 months in age (Korean Native Cattle) at three different farms, which were located in different provinces in Korea. Forty-five cattle were raised with feed supplemented with 0.5% germanium biotite (SoltoB, Seobong BioBestech Co, Seoul, Korea; germanium biotite group) from 1 week before vaccination to the end of the experiment, while the rest were raised without supplementation (control group). Control and germanium biotite groups were arranged at each farm (Table 3). All the cattle were intramuscularly vaccinated with 2 ml of Decivac FMD DOE trivalent vaccine (InterVet, Germany). A booster vaccination was given 4 weeks after the first vaccination, according to the national vaccination policy of Korea. Samples of blood, saliva, and feces were collected before vaccination and up to 6 months after the second vaccination. Feces and saliva were collected by swabbing with a cotton swab. Bleeding was carried out from the jugular vein. Sera were collected and stored at −20°C until use after inactivation at 56°C for 30 min. The cotton swabs were immersed in 1 ml of PBS for 1 h at 4°C, and the supernatants were collected after centrifugation at 1,500 × g for 15 min at 4°C; the supernatants were stored at −20°C and used as samples for the ELISA analysis of FMDV-specific IgA.

**Table 3:** Experiment design for grouping, sampling, and analysis

**Farms**	**Number of cattle**		**Samples**	**Analysis of**
	**Control**	**Germanium biotite**		
A	26	22	Serum, saliva, feces	Inhibition percentage, ELISA for antibody levels (IgG and IgA), lysozyme activity
B	13	18	Serum, saliva, feces	Inhibition percentage, ELISA for antibody levels (IgG and IgA), lysozyme activity, virus neutralization test
C	5	5	Serum, saliva, feces, PBMC	Inhibition percentage, ELISA for antibody levels (IgG and IgA), lysozyme activity, PBMC (phenotype, cytokines, proliferation)

Among 89 cattle at 6–8 months of age, 45 cattle were raised with feeds supplemented with 0.5% germanium biotite from 1 week before vaccination to the end of experiment (germanium biotite group) and the rest of them were raised without supplementation (control group).

The germanium biotite consisted of silicon dioxide (61.90%), aluminum dioxide (23.19%), iron oxide (3.97%), sodium oxide (3.36%), calcium oxide (<2%), magnesium oxide (<2%), titanium oxide (<2%), and 36 ppm germanium. All procedures in the animal experiment in this study were approved by the Institution Animal Care and Use Committee (IACUC) of Seoul National University (SNU-111210-1).

### 5.2 Measurement of antibody against FMDV

The antibody titers against FMDV were measured using the PrioCHECK FMDV type O ELISA kit (Prionics, Switzerland) following the manufacturer’s protocols. The serotype O antibody titer using this ELISA kit is the criterion for assessing seropositivity in the national policy of Korea. The antibody titer against FMDV was indicated by the PI value. The high PI value presents a high level of antibody titer, and the sera that showed more than 50% of the PI value were considered to be seropositive in the ELISA assays. FMDV-specific IgG in the serum were measured using the PrioCHECK FMDV type O ELISA kit (Prionics) with 1:40 diluted serum and horseradish peroxidase (HRP)-conjugated sheep anti-bovine IgG (Bethyl, USA). In addition, FMDV-specific IgA in the saliva and feces was measured using the PrioCHECK FMDV type O ELISA kit (Prionics) with 1 x samples extracted by PBS from cotton swabs and HRP-conjugated rabbit anti-bovine IgA (Bethyl). All procedures for detecting FMDV-specific IgG and IgA followed the manufacturer’s protocols with a 1:3,000 dilution of each immunoglobulin-specific conjugate.

### 5.3 Analysis of VN antibody titer

The VN titer was measured in eight randomly selected sera from the germanium biotite and seven sera from the control groups of Farm B at 20 and 31 weeks after vaccination (Tables 1 and 3). Fifty microliters of twofold serially diluted bovine sera were reacted with 50 μl of the FMDV Andong strain (100TCID_50_), a serotype O FMDV Korean isolate from the 2010 outbreak, at 37°C for 1 h. After incubation, 50 μl of complete Dulbecco’s Modified Eagle Medium (Gibco, USA) with 5% fetal bovine serum containing 1 × 10^4^ LFBK cells/ml was added to the reaction mixture. After incubation of the reaction mixtures at 37°C for 48 h, the VN titer was determined as the final dilution of serum showing no cytopathic effect.

### 5.4 Analysis of PBMC subsets

Among the 89 cattle, analysis of the peripheral lymphocyte profiles was conducted with ten Korean native cattle (IACUC permission no. SNU-111210-1). Five of them were fed with a commercial feed supplemented with 0.5% germanium biotite (germanium biotite group), while the rest were fed without the supplement (control group). PBMCs were cultured from the heparinized whole blood of the experiment cattle using the Histopaque®-1077 Hybri-Max™ (Sigma, USA) system, as described by the manufacturer. The PMBCs were then stimulated with DPBS (Gibco), ConA (5 g/ml, Sigma) for CD3, CD4, CD8, and MHC I and II and LPS (5 g/ml, Sigma) for CD79a staining for 96 h to characterize the phenotype of PBMC responding to stimulation with mitogens. The procedures for cell fixation and antibody reaction to detect the cell markers were performed as previously reported [37] using the DPBS with 0.2% Tween® 20 (Sigma) as the permeabilisation solution. To prevent a non-specific reaction of the Fc receptor with the detection antibodies, the fixed cells were reacted with normal Ig from the antibody-producing host for 10 min and reacted with fluorescein isothiocyanate (FITC) or phycoerythrin (PE)-conjugated cell surface marker detection antibodies. After the reaction, the number of cells reacting with the FITC or PE conjugate was analyzed using Flow cytometric analysis (BD, USA). The monoclonal antibodies used for the staining of the cells surface markers are listed in Table 4.

**Table 4:** Antibodies used to detect the cell surface markers

**Target**	**Antibody**	**Company**
CD79a	PE-conjugated mouse anti-bovine CD79a	Abcam, UK
CD3	FITC-conjugated hamster anti-bovine CD3	Abcam
CD4	PE-conjugated mouse anti-bovine CD4	US Biological, USA
CD8	PE-conjugated mouse anti-bovine CD8	LifeSpan BioSciences, USA
MHC Ι	FITC-conjugated mouse anti-bovine MHC Ι	Novus Biological, USA
MHC ΙΙ	Mouse anti-bovine MHC ΙΙ^a^	Abcam
Fc receptor blocking	Polyclonal mouse IgG fraction	Abcam
	Monoclonal Armenian hamster IgG fraction	Abcam

^a^Mouse anti-bovine MHC ΙΙ antibody was conjugated with PE using an EasyLink B-Phycoerythrin conjugation kit (Abcam).

### 5.5 Analysis of cytokine gene expression in PBMC

Total RNAs were extracted from PBMCs with the RNeasy mini kit (Qiagen, USA), and cDNAs were synthesized with the reverse transcription kit (Qiagen). The relative expression levels of IFN-γ, IL-1α, IL-1β, IL-4, IL-6, and IL-10 were quantitated using the Rotor-Gene SYBR Green PCR kit (Qiagen) and Rotor-Gene Q (Qiagen), as previously reported [38]. The expression levels were normalized by β-actin expression using the 2^-∆∆C^_T_ method [39].

### 5.6 Analysis of PBMC proliferation

A lymphocyte proliferation assay was conducted with the PBMCs collected from the experiment cattle. Ninety microliters of PBMCs (5 × 10^6^ cells/ml) in a 96-well plate were incubated with ConA (5 μg/ml) and LPS (5 μg/ml) at 37°C for 96 h in a 5% CO_2_ atmosphere. Absorbance at 560 nm was measured 2 h after the addition of 10 μl of 10× Prestoblue (Invitrogen, USA). Proliferation of the PBMCs was compared based on this absorbance.

### 5.7 Analysis of lysozyme activities

Lysozyme activities in the serum were measured based on the dissolution ability against *Micrococcus lysodeikticus* (Sigma) [40]. The activity was calculated based on the standard curve generated from the serially diluted lysozyme solution.

### 5.8 Statistical analysis

The data were expressed as mean ± standard deviation (SD). A student’s *t*-test and repeated measures of ANOVA were performed for statistical analysis of the data. All statistical analyses of data were carried out using SPSS version 19.0 software (SPSS, USA). A probability value of *p* < 0.05 was considered significant.

## Abbreviations

ConA: Concanavalin A

FITC: Fluorescein isothiocyanate

FMD: Foot-and-mouth disease

FMDV: Foot-and-mouth disease virus

HRP: Horseradish peroxidase

IACUC: Institution Animal Care and Use Committee

LPS: Lipopolysaccharide

MHC: Major histocompatibility complex

PBMC: Peripheral blood mononuclear cell

PE: Phycoerythrin

PI: Inhibition percentage

SAT: South African territories

Th1: T helper type 1

Th2: T helper type 2

VN: Virus neutralization

## Competing interests

Effects of the germanium biotite in FMD vaccination are applying to patent in Korea.

## Authors’ contributions

MJ was involved in sampling, sample processing, data analysis and drafting of manuscript. MKS was involved in sampling and participated in the studies of ELISA for antibodies. SBC and SWS carried out sampling and participated in study of cytokine. AY carried out sampling. WJL and HTP participated in the analysis of PBMC and in study of cytokines analysis. JHP and BK carried out virus neutralization test with a Korea isolate of FMDV. YKJ carried out development of germanium biotite. HSY was involved in experimental design and supervision of the experiments and manuscript revision. All authors read and approved the final manuscript.
